# Exploring approaches to tackle cross-domain challenges in brain medical image segmentation: a systematic review

**DOI:** 10.3389/fnins.2024.1401329

**Published:** 2024-06-14

**Authors:** Ming Yanzhen, Chen Song, Li Wanping, Yang Zufang, Alan Wang

**Affiliations:** ^1^School of Artificial Intelligence Academy, Wuhan Technology and Business University, Wuhan, Hubei, China; ^2^Institute of Information and Intelligent Engineering Applications, Wuhan Technology and Business University, Wuhan, Hubei, China; ^3^Wuhan Dobest Information Technology Co., Ltd, Hubei, China; ^4^Auckland Bioengineering Institute, The University of Auckland, Auckland, New Zealand; ^5^Medical Imaging Research Center, Faculty of Medical and Health Sciences, The University of Auckland, Auckland, New Zealand; ^6^Centre for Co-Created Ageing Research, The University of Auckland, Auckland, New Zealand; ^7^Centre for Brain Research, The University of Auckland, Auckland, New Zealand

**Keywords:** brain medical image, segmentation, cross-domain, stroke, white matter, brain tumor, normalization

## Abstract

**Introduction:**

Brain medical image segmentation is a critical task in medical image processing, playing a significant role in the prediction and diagnosis of diseases such as stroke, Alzheimer's disease, and brain tumors. However, substantial distribution discrepancies among datasets from different sources arise due to the large inter-site discrepancy among different scanners, imaging protocols, and populations. This leads to cross-domain problems in practical applications. In recent years, numerous studies have been conducted to address the cross-domain problem in brain image segmentation.

**Methods:**

This review adheres to the standards of the Preferred Reporting Items for Systematic Reviews and Meta-Analyses (PRISMA) for data processing and analysis. We retrieved relevant papers from PubMed, Web of Science, and IEEE databases from January 2018 to December 2023, extracting information about the medical domain, imaging modalities, methods for addressing cross-domain issues, experimental designs, and datasets from the selected papers. Moreover, we compared the performance of methods in stroke lesion segmentation, white matter segmentation and brain tumor segmentation.

**Results:**

A total of 71 studies were included and analyzed in this review. The methods for tackling the cross-domain problem include Transfer Learning, Normalization, Unsupervised Learning, Transformer models, and Convolutional Neural Networks (CNNs). On the ATLAS dataset, domain-adaptive methods showed an overall improvement of ~3 percent in stroke lesion segmentation tasks compared to non-adaptive methods. However, given the diversity of datasets and experimental methodologies in current studies based on the methods for white matter segmentation tasks in MICCAI 2017 and those for brain tumor segmentation tasks in BraTS, it is challenging to intuitively compare the strengths and weaknesses of these methods.

**Conclusion:**

Although various techniques have been applied to address the cross-domain problem in brain image segmentation, there is currently a lack of unified dataset collections and experimental standards. For instance, many studies are still based on n-fold cross-validation, while methods directly based on cross-validation across sites or datasets are relatively scarce. Furthermore, due to the diverse types of medical images in the field of brain segmentation, it is not straightforward to make simple and intuitive comparisons of performance. These challenges need to be addressed in future research.

## 1 Introduction

**Figure 1:**
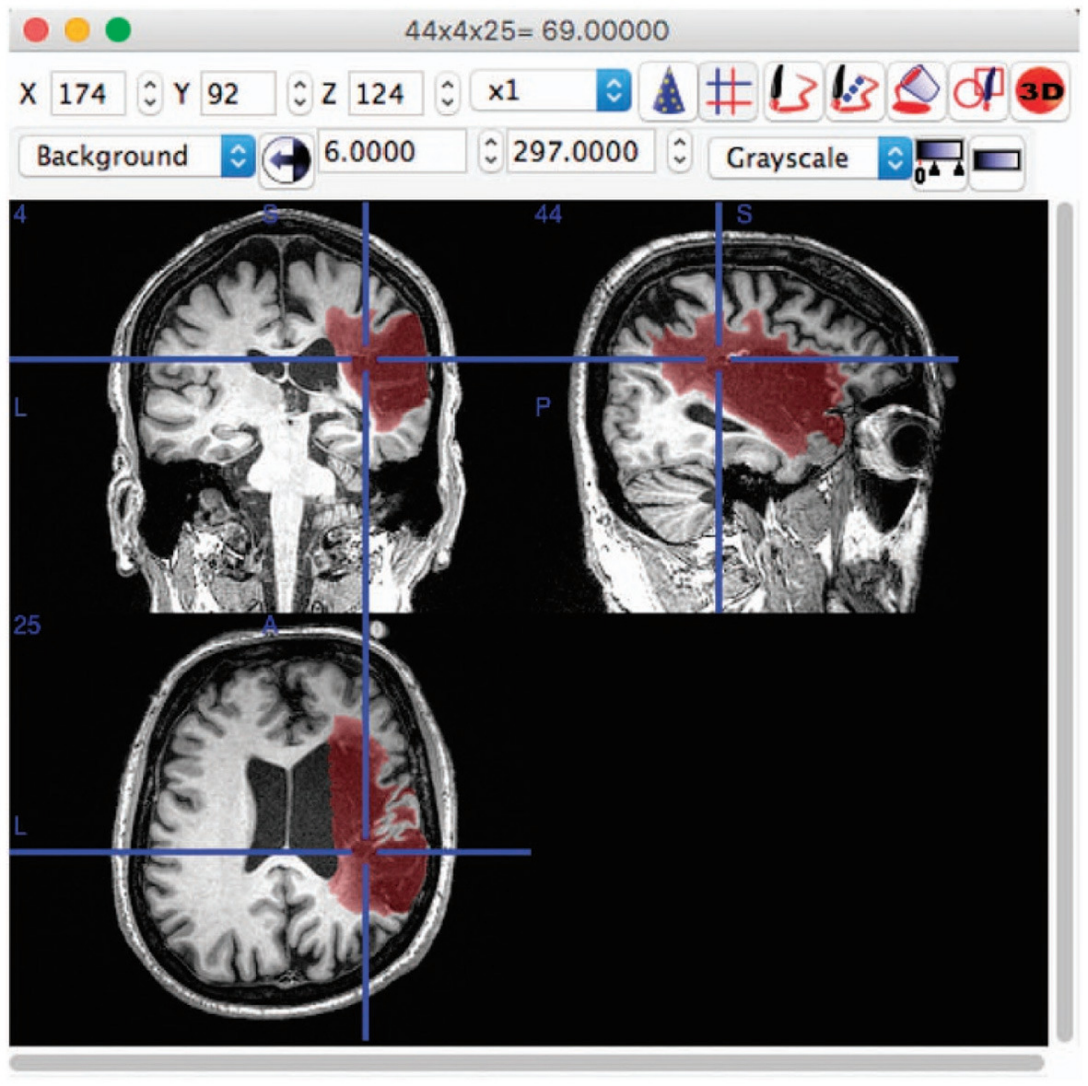
An example of lesion segmentation in brain (Liew et al., 2017).

Medical image segmentation, particularly for the brain, is a crucial and challenging task in the field of medical imaging analysis, with a wide range of applications from disease diagnosis to treatment planning. The complexity of this task is further compounded when considering the cross-domain nature of the data, arising from variations in scanners, imaging protocols, and patient populations among different sites (Dolz et al., 2018; Ravnik et al., 2018). This review aims to provide an overview of the progress made in the domain of cross-domain brain medical image segmentation. As depicted in Figure 1, the brain images and the corresponding segmented lesion areas are illustrated.

Domain-adaptive methods are designed to adapt a model that has been trained on one domain (the source domain) to perform well on a different, but related domain (the target domain). This is useful in situations where we have a lot of labeled data in the source domain but little to no labeled data in the target domain. Domain adaptation techniques attempt to learn the shift or differences between the source and target domains and adjust the model accordingly. Techniques can include feature-level adaptation, instance-level adaptation, and parameter-level adaptation, among others.

Non-adaptive methods, on the other hand, do not make any adjustments to account for differences between the source and target domains. They are trained on one domain and then directly applied to another. This approach can work well if the source and target domains are very similar, but performance can degrade if there are significant differences between the two domains. Non-adaptive methods do not leverage any domain adaptation techniques and hence, can suffer from a problem known as domain shift or dataset shift, where the distribution of data in the target domain differs from the distribution in the source domain.

The advent of deep learning methods, especially Convolutional Neural Networks (CNNs) (LeCun et al., 1998) and their variants, has significantly improved the performance of image segmentation tasks (Dolz et al., 2018; Ravnik et al., 2018; Huang et al., 2020; Liu Y. et al., 2020). However, these models often suffer from limited generalization capability when applied to unseen data from different domains (Knight et al., 2018; Bermudez and Blaber, 2020; Zhou et al., 2022). To address this, various domain adaptation techniques have been proposed, including transfer learning, unsupervised learning, and self-supervised learning (Knight et al., 2018; Atlason et al., 2019; Ntiri et al., 2021; Tomar et al., 2022).

Transfer learning has emerged as a popular approach to leverage pre-trained models on new data, demonstrating success in various studies (Knight et al., 2018; Bermudez and Blaber, 2020; Zhou et al., 2022; Liu D. et al., 2023; Torbati et al., 2023). Unsupervised learning methods, which do not require labeled data from the target domain, have also shown promising results in cross-domain brain image segmentation (Atlason et al., 2019; Rao et al., 2022). Recently, self-supervised learning, where models are pre-trained on auxiliary tasks before being fine-tuned on the main task, has been increasingly adopted (Ntiri et al., 2021; Liu et al., 2022a; Tomar et al., 2022).

Besides, different strategies have been proposed to handle specific challenges in cross-domain brain image segmentation. For instance, normalization techniques have been used to reduce the scanner-related variability (Ou et al., 2018; Goubran et al., 2020; Dinsdale et al., 2021). Generative Adversarial Networks (GANs) (Goodfellow et al., 2014) have been employed to generate synthetic images that share the same distribution as the target domain, thus improving the model's generalizability (Zhao et al., 2019; Cerri et al., 2021; Tomar et al., 2022). Model ensembling and federated learning approaches have also been explored to leverage the strengths of multiple models or to perform decentralized learning (Reiche et al., 2019).

Moreover, the application of advanced architectures, such as 3D-CNNs (Ji et al., 2013), Transformers (Vaswani et al., 2017), and UNets, has further enhanced the performance of brain image segmentation across different domains (Dolz et al., 2018; Goubran et al., 2020; Huang et al., 2020; Liu Y. et al., 2020; Basak et al., 2021; Li et al., 2021; Meyer et al., 2021; Sun et al., 2021; Zhao et al., 2021). These models have been applied to various brain structures and conditions, including white matter, brain tumors, multiple sclerosis, and stroke (Erus et al., 2018; Knight et al., 2018; Ravnik et al., 2018; Reiche et al., 2019; Basak et al., 2021; Jiang et al., 2021; Kruger et al., 2021; Li et al., 2021; Sun et al., 2021; Kaffenberger et al., 2022; Zhou et al., 2022; Liu D. et al., 2023; Yu et al., 2023b; Zhang et al., 2023).

Despite the significant progress, cross-domain brain image segmentation remains a challenging problem. Future research directions may include the development of more robust and generalizable models, the exploration of novel domain adaptation techniques, and the incorporation of multimodal imaging data to improve segmentation performance. The studies reviewed herein provide valuable insights into these potential avenues for future advancement (Liu Y. et al., 2020; Jiang et al., 2021; Liu et al., 2022a; Rao et al., 2022; Torbati et al., 2023).

## 2 Materials and methods

### 2.1 Inclusion criteria and search terms

The search process for this study adheres to the Preferred Reporting Items for Systematic Reviews and Meta-Analyses (PRISMA) (Moher et al., 2009) guidelines. In order to gather relevant research on cross-domain issues in brain medical image segmentation, we have designated three main categories of keywords: Medical Imaging, Segmentation, and Domain. Specific keywords for each category are shown in Table 1. It's worth noting that we use the Boolean operator “OR” to connect keywords within the same category, while “AND” is used to connect different categories. This way, we can construct complex search queries. Because the focus of the research is on cross-domain issues in brain medical image segmentation, these articles will be included in our review.

**Table 1:** Search terms used for the electronic databases.

**Category**	**Keywords**
Medical image	Medical, biomedical, semantic, neurological, brain, MRI, CT
Segmentation	Segmentation, thresholding, region growing, edge detection, level set method, clustering, graph cut, U-Net, Mask R-CNN
Domain	Different scanners, different sites, cross-domain, cross-platform, unseen datasets, multiCenter, multi-site, multi-scanner, harmonization, normalization, leave-one-site-out

### 2.2 Screening and selection process

We used three search engines for literature retrieval: PubMed, IEEE, and Web of Science, with the search time frame being from January 2018 to December 2023 for journal or conference articles. In compliance with the PRISMA guidelines, the first stage of the screening process is to merge duplicate articles from different search engines. In the second stage, we screen based on the title and abstract of the articles, discarding those not relevant to our discussion topic, such as those that do not include keywords like “brain medical imaging,” “segmentation,” or “domain” in the title and abstract. In the third stage, we filter out eligible articles through a full-text review. Reasons for exclusion may include: inability to access the full text; non-English articles; survey studies or literature reviews; non-original research; not focusing on cross-domain issues; not describing experiments or validation studies; not using multi-site or multi-scanner datasets.

### 2.3 Data extraction

From the screened articles, we extracted the following information: author names, publication year, dataset name, dataset size, parts included in the dataset, cross-domain type, solution method, and evaluation metrics. For more detailed information about solution method, please refer to Tables 2, 3.

**Table 2:** Category of solution method.

**Category**	**Solution method**	**Description**
Neural network	UNet, CNN, 3D-CNN, Transformer, GAN, model ensembling	Different structures of neural networks optimized for learning from data, especially high-dimensional data like images
Learning types	Supervised, Self-supervised, unsupervised	Strategies for training models, varying by how they use labeled or unlabeled data
Learning strategies	Transfer learning, incremental learning, federated learning	Techniques to improve model training, often leveraging pre-existing knowledge, adapting over time, or distributing the learning process
Mathematical methods	Bayesian, Fourier, Logistic Regression	Use of specific mathematical techniques to provide theoretical foundations, handle uncertainty, or offer interpretability
Data preprocessing techniques	Data augmentation, normalization, FLAIR	Steps to improve data quality, variety, or scale before inputting it into a model
Tools	iBEAT V2.0, FreeSurfer, LST	Automatic segmentation toolkit, advanced algorithms, user-friendly interfaces

**Table 3:** Key features of solution method.

**Solution method**	**Key features**	**Advantages**	**Disadvantages**
UNet	Biomedical image segmentation	Excellent on small medical datasets	May overfit on small datasets
CNN	Visual data analysis	Good performance on large, labeled image datasets	Requires large amounts of data and computational resources
3D-CNN	3D spatial relationships	Superior on 3D medical imaging datasets	Requires larger computational resources and data
Transformer	Self-attention mechanisms	Handles long-range dependencies, parallelizable	Computationally intensive, needs tuning
GAN	Data generation	Augments existing data, improves model robustness	Training can be unstable and difficult
Model Ensembling	Combines multiple models	Leverages strengths of each model, improves performance	Increases computational complexity
Supervised	Learns from labeled data	High performance on large labeled datasets	Requires labeled data, expensive to collect
Self-supervised	Creates learning task from data itself, such as Masked Image Modeling	Efficient use of data, learns better feature representations, supports pre-training and fine-tuning	Performance may be lower than supervised methods
Unsupervised	Learns from unlabeled data, such as K-means	No need for labels, discovers unknown patterns, suitable for anomaly detection	Learned features may not be task-specific
Transfer learning	Uses pre-trained model	Reduces need for data and computational resources	Pre-trained model may require adjustments
Incremental learning	Gradual learning over time	Adapts to new data over time, less memory-intensive	Sensitive to data order, may forget old data
Federated Learning	Trains across multiple decentralized devices	Preserves privacy, learns from distributed data	Requires careful coordination, faces data heterogeneity issues
Bayesian	Provides measure of uncertainty	Important in medical applications for risk assessment	Computationally intensive, needs careful design of prior
Fourier	Transforms data into different domain	Reveals periodic patterns, filters noise	May lose spatial information
Logistic regression	Used for binary classification tasks	Simple, fast, interpretable results	May struggle with complex tasks
Data augmentation	Increases amount of training data	Improves model performance and robustness	Augmented data may not cover all possible variations
Normalization	Adjusts values to a common scale	Improves performance, reduces influence of outliers	May lose information about original scale
FLAIR	High-contrast images	Suppression of cerebrospinal fluid signals	Sensitive to magnetic field inhomogeneities
iBEAT V2.0	Comprehensive processing and analysis of brain MRI data	User-friendly interface, comprehensive solution	Requires substantial computational resources, steep learning curve
FreeSurfer	Comprehensive processing and analyzing of brain MRI data	High-quality cortical surface reconstructions, quantification of brain structures	Long execution time, steep learning curve
LST	Automatic segmentation	handling multi-modal MRI data	Performance influenced by image quality and lesion type

Enhancements based upon the UNet model continue to represent a prevalent research direction in medical image segmentation. Subsequent models, such as 3D-CNN, exhibit commendable performance in many 3D data scenarios, albeit at the cost of requiring substantial computational resources. In comparison, newer network structures like Transformer are gradually gaining traction in the field of medical segmentation, and it is anticipated that a plethora of innovations will be spawned from this methodology.

Methods grounded in different learning types are somewhat niche in comparison. On the whole, the outcomes of unsupervised and semi-supervised learning methods are not as effective as their supervised counterparts. This discrepancy is likely attributable to the relatively smaller datasets available in the field of medical imaging, unlike the voluminous data present in natural language processing and computer vision.

Mathematically-based methods are currently often amalgamated with deep learning models to enhance their interpretability. This area of work is particularly meaningful and holds significant potential.

There is a broad spectrum of data preprocessing techniques available, including Generative Adversarial Networks (GANs), which can be employed for data augmentation to enhance data diversity.

The array of tools available for medical image segmentation is continually expanding, and the barriers to their utilization are concurrently lowering.

In addition to extracting key data from cross-domain research in the field of brain image segmentation, we have also conducted a focused comparative analysis of cross-domain algorithms for three important branches of brain image segmentation: stroke lesion segmentation, white matter segmentation and brain tumor segmentation.

Due to the variety of datasets employed in the selected articles, it is challenging to compare the merits and demerits of each algorithm on a holistic basis. To compare the effectiveness of these algorithms, it becomes necessary to delve into more specific areas of segmentation. The ATLAS, MICCAI 2017 and BraTS datasets, each employed five times, stand out as the most frequently used. They correspond respectively to stroke lesion segmentation, white matter segmentation and brain tumor segmentation.

## 3 Results

**Figure 2:**
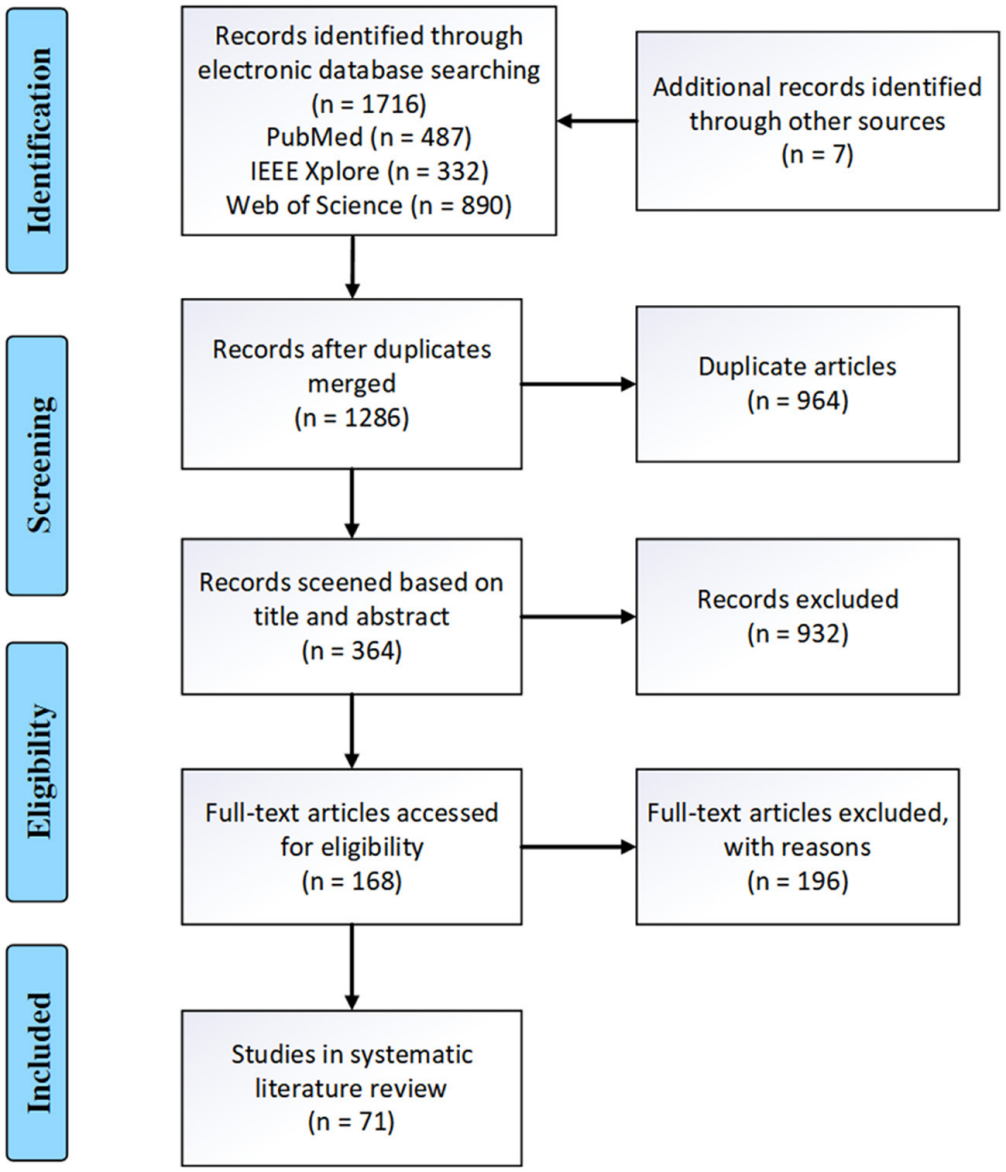
The PRISMA diagram detailing this systematic review.

Figure 2 presents the PRISMA flow diagram for this task. The number of articles from the three databases (PubMed, IEEE, Web of Science) were 487, 332, and 890 respectively. An additional seven articles were identified through the references of confirmed papers. After merging duplicate studies, 1,286 articles were obtained. Following the title and abstract screening, 364 articles remained. Finally, after full-text review, 71 articles were included for publication. Table 4 documents the details of the finally collected articles.

**Table 4:** A summary of the data extracted from the reviewed papers.

**Paper**	**Dataset name**	**Disease or region**	**MRI or CT**	**Public or private**	**Data number**	**Cross-domain type**	**Solution method**	**Evaluation metrics**
Ravnik et al. (2018)		White matter	MRI	Private	60	Multi-scanner	CNN	DSC, TPR
Dolz et al. (2018)	ISBR, ABIDE	Brain	MRI	Public	1,157	Multi-site	3D-CNN	DSC, MHD
Knight et al. (2018)	MICCAI 2017, MICCAI 2016, ISBI MS 2015	White matter	MRI	Public	96	Multi-scanner	Logistic regression	Similarity Index, precision, recall
van Opbroek et al. (2018)	HarP, RSS	Hippocampus	MRI	Public	135	Multi-scanner	Transfer learning	DSC
Karani et al. (2018)	HCP, ABIDE, ADNI, IXI(D5)	Brain	MRI	Public		Multi-scanner	UNet	DSC
Doyle et al. (2018)		MS	MRI	Private	798	Multi-site	Bayesian	Sensitivity, specificity
Goubran et al. (2020)		Hippocampal	MRI	Private	509	Multi-center	3D-CNN	DSC, Jaccard
Zhao et al. (2019)		Infant brain	MRI	Private	233	Mulit-site	GAN	MAE, PSNR
Bui and Wang (2019)		Infant brain	MRI	Private		Mulit-site	3D-CNN	DSC, 95HD
Reiche et al. (2019)		White matter	MRI	Private		Multi-center	Normalization	DSC, HD, sensitivity
Jiang et al. (2021)		Brain	CT	Private	10	Multi-modal	Transfer learning	NMI, ARI
Erus et al. (2018)	BLSA	Brain	MRI	Public	721	Mulit-site	Label fusion	ICC
McClure et al. (2019)	NNDSP	Brain	MRI	Public		Mulit-site	Bayesian	DSC
Zhang et al. (2019)	MICCAI 2017	White matter	MRI	Public	170	Multi-site	UNet	DSC
Fung et al. (2019)		Hippocampal	MRI	Private	27	Multi-scanner	Freesurfer	ICC
Khademi et al. (2020)	CAIN, ADNI	Brain	MRI	Public		Multi-center	Normalization	DSC
Dewey et al. (2019)		MS	MRI	Private	55	Multi-scanner	UNet	DSC, PVD
Nair et al. (2020)		MS	MRI	Private	1,064	Multi-site	3D-CNN	TPR, FDR
Le et al. (2019)		MS	MRI	Private	87	Multi-center	FLAIR	LVD, DSC, sensitivity, SSD
Liu Y. et al. (2020)		Brain	MRI	Private	36	Multi-center	3D-CNN	DSC, ASSD
Dinsdale et al. (2020)	OASIS, UK Biobank	Brain	MRI	Public		Multi-site	UNet	DSC
Billast et al. (2019)		MS	MRI	Private	410	Multi-scanner	CNN	DSC, precision, recall
Ou et al. (2018)		Brain	MRI	Private	126	Mulit-site	Normalization	DSC
Dinsdale et al. (2021)	OASIS, UK Biobank	Brain	MRI	Public		Mulit-site	CNN	DSC
Cerri et al. (2021)	MSSeg, Trio, Achieva, ISBI	MS	MRI	Public	119	Multi-site	Model ensembling	DSC, precision, recall
Bermudez and Blaber (2020)		Brain	MRI	Private	111	Multi-site	Data augmentation	DSC
Borges et al. (2019)	SABRE	Brain	MRI	Private	22	Mulit-site	UNet	DSC
Monteiro et al. (2020)		Brain	CT	Private	538	Multi-center	CNN	DSC
Huang et al. (2020)	ATLAS	Stroke	MRI	Public	304	Mulit-site	UNet	DSC, precision, recall
Brown et al. (2020)		Hippocampal	MRI	Private		Multi-scanner	Freesurfer	ICC
Kim et al. (2020)	Multicenter, RM, CND	Brain	MRI	Public and private		Multi-center	UNet	ICC
Liu S. et al. (2020)		Brain	MRI	Private	15	Multi-scanner	Freesurfer	CV
Srinivasan et al. (2020)	EADC-ADNI, ADNI	Infant brain	MRI	Public		Multi-site	Freesurfer	ROI volumes
Basak et al. (2021)	ATLAS	Stroke	MRI	Public	304	Mulit-site	3D-CNN	DSC, precision, recall
Sun et al. (2021)	BCP	Infant brain	MRI	Private	160	Multi-site	Self-supervised	DSC
Ntiri et al. (2021)		Cerebrovascular	MRI	Private	238	Multi-site	3D-CNN	DSC, Jaccard, HD, processing time
Niu et al. (2022)		Brain	MRI	Private	48	Multi-scanner	GAN	Test-retest variability (TRV)
Zhao et al. (2021)	MICCAI 2017	White matter	MRI	Public + private	170+	Mulit-site	UNet	Score (F1)
Meyer et al. (2021)	CNNOASIS, CNNOASIS-DA, MS	MS	MRI	Public		Multi-scanner	Data augmentation	DSC
Kushibar et al. (2021)	IBSR, MICCAI 2012, MICCAI 2017	Brain	MRI	Public and private		Multi-center	Transfer learning	DSC
Sundaresan et al. (2021)	NDGEN, OXVASC	White matter	MRI	Public	39	Multi-scanner	Transfer learning	DSC
Kruger et al. (2021)		MS	MRI	Private	1809	Multi-scanner	CNN	Sensitivity
Li et al. (2021)	NeoBrainS12, dHCP	Neonatal brain	MRI	Public	47	Multi-modal	GAN	DSC
Ribaldi et al. (2021)		White matter	MRI	Private	53	Multi-site	LST	DSC
Goodkin et al. (2021)		White matter	MRI	Private	66	Multi-center	FLAIR	DSC
Memmel et al. (2021)	MSD, Scientific Data	Hippocampal	MRI	Public	195	Multi-site	GAN	DSC
Kamraoui et al. (2022)	ISBI, MICCAI 2016	MS	MRI	Public and private		Multi-site	3D-CNN	DSC
Zhou et al. (2022)	ATLAS	Stroke	MRI	Public	304	Mulit-site	Self-supervised	DSC, precision, recall
Tomar et al. (2022)	CANDI, OASIS	Brain	MRI	Public	131	Multi-site	Self-supervised	DSC
Opfer et al. (2023)	IBSR, FTHP	Thalamus	MRI	Public+ private	127	Multi-scanner	3D-CNN	DSC
Liu et al. (2022a)	BraTS 2018	Brain tumor	MRI	Public	285	Multi-modal	Unsupervised	DSC, HD
Wang Y. et al. (2022)	ECHO, M-CRIB	Infant brain	MRI	Public	473	Multi-scanner	Transfer learning	DSC, ICC, ASD
Kaffenberger et al. (2022)		Stroke	CT+ MRI	Private	50	Multi-modal	Normalization	DSC, HD
Trinh et al. (2022)	iSeg-2017	Infant brain	MRI	Public	23	Multi-site	UNet	DSC, MHD, ASD
Rao et al. (2022)	DLBS, SALD, IXI, COBRE	Brain	MRI	Public		Mulit-site	Transformer	DSC, Jaccard Index, HD
Kalkhof et al. (2022)	MSD	Hippocampal	MRI	Public	260	Multi-site	GAN	DSC
Torbati et al. (2023)		Brain	MRI	Private	18	Multi-scanner	Supervised	GM-WM, segmentation similarity
Zhang et al. (2023)	Heckto, BraTS 2018	Brain	MRI	Public	411	Multi-modal	Self-supervised	DSC, sensitivity
Yu et al. (2023a)	ATLAS	Stroke	MRI	Public	304	Mulit-site	Normalization	DSC, Recall
Han et al. (2023)	ADNI, EMCI	Brain	MRI	Public	391	Multi-scanner	Transformer	Acc,IoU
Hindsholm et al. (2023)		MS	MRI	Private	746	Multi-scanner	UNet	DSC, precision, recall
Liu X. et al. (2023)		Brain tumor	MRI	Private	285	Multi-site	Incremental learning	DSC, HD
Kazerooni et al. (2023)		Brain tumor	MRI	Private	244	Multi-center	3D-CNN	DSC
Yu et al. (2023b)	ATLAS	Stroke	MRI	Public	304	Mulit-site	Fourier	DSC, precision, recall
Liu D. et al. (2023)	MICCAI 2016	MS	MRI	Public + private	188	Multi-site	Federated learning	DSC, TPR, FPR
Zuo et al. (2023)	OASIS3, BLSA	White matter	MRI	Public and private		Multi-site	UNet	DSC
Wang et al. (2023)	BCP, dHCP, MSMS6	Infant brain	MRI	Private		Multi-site	iBEAT V2.0	DSC, ASD
Park et al. (2021)	MICCAI 2017	White matter	MRI	Public		Multi-site	UNet	DSC, precision, recall
Qin et al. (2023)	BraTS 2019	Brain tumor	MRI	Public		Multi-site	Unsupervised	DSC
Tomar et al. (2021)	BraTS 2015, WHSD	Brain tumor	CT + MRI	Public		Multi-modal	Normalization	DSC
Liu et al. (2022b)	BraTS 2018	Brain tumor	MRI	Public		Multi-site	Unsupervised	DSC

### 3.1 Year of publication

**Figure 3:**
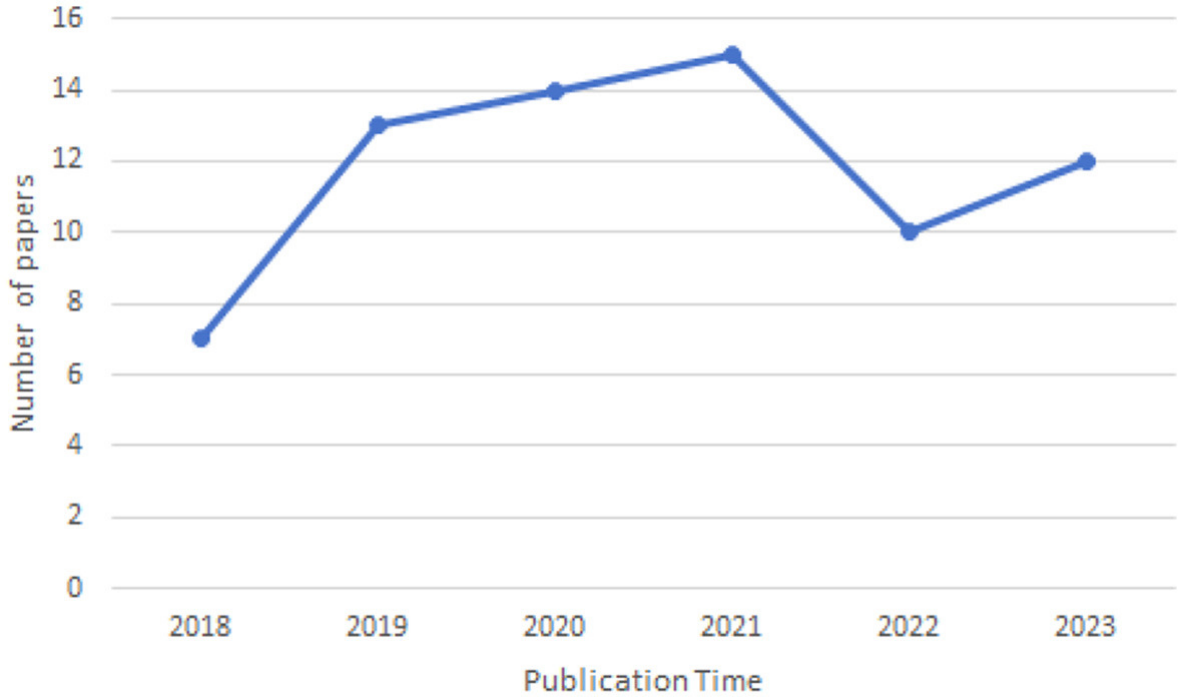
Year of publication of the reviewed papers.

As illustrated in the Figure 3, the number of papers addressing cross-domain segmentation in brain imaging has been increasing annually from 2018 to the present, with a peak of 15 papers in 2021. This trend indicates that there are still many challenges to overcome in this field, affirming its status as an active area of research.

### 3.2 Datasets

**Figure 4:**
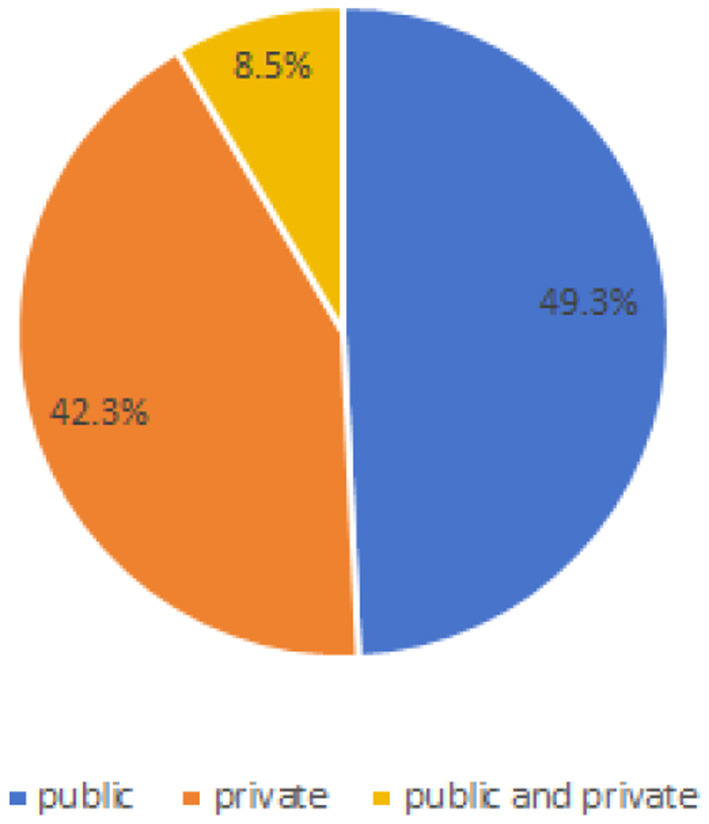
Proportion of public or private.

**Figure 5:**
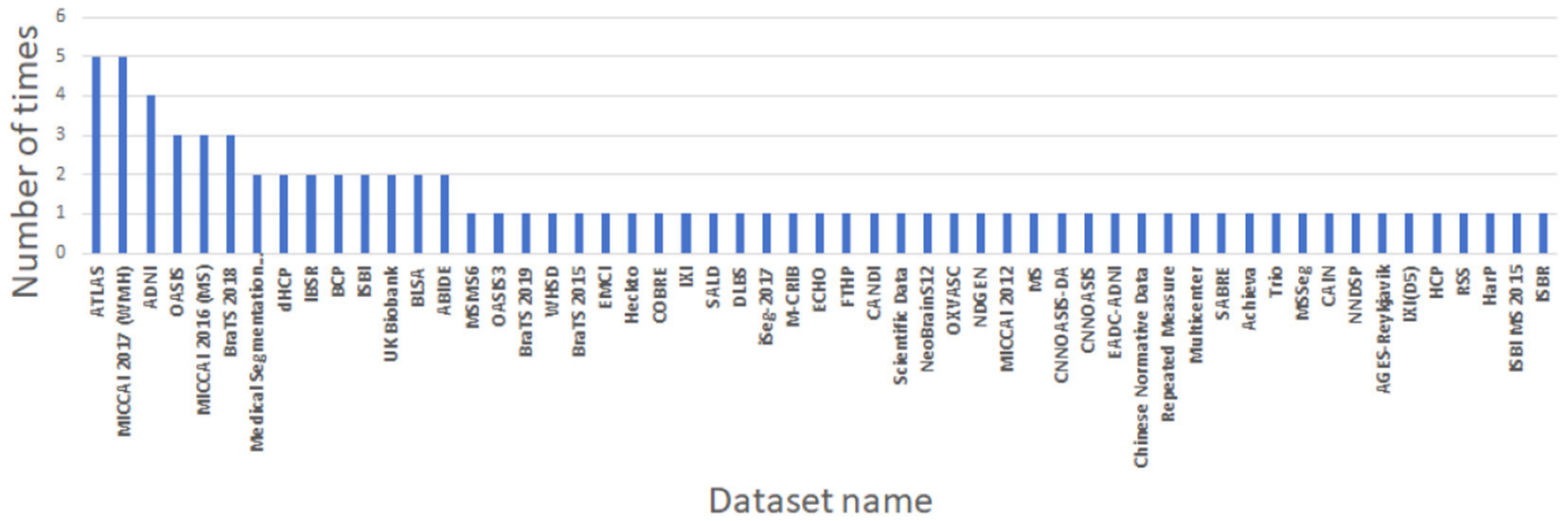
Information of datasets used by the reviewed papers.

As can be seen from Table 4 and Figure 4, in the 71 articles reviewed, 41 utilized public datasets, encompassing 56 different types. Among these, from Figure 5, the most frequently used datasets were ATLAS, MICCAI 2017 and BraTS, only five times. The remaining datasets were used less, with the majority being used only once. Thus, within the field of brain image segmentation, many articles addressing cross-domain issues still rely on proprietary datasets, and those that do use public datasets draw from a wide variety.

### 3.3 Disease or region

**Figure 6:**
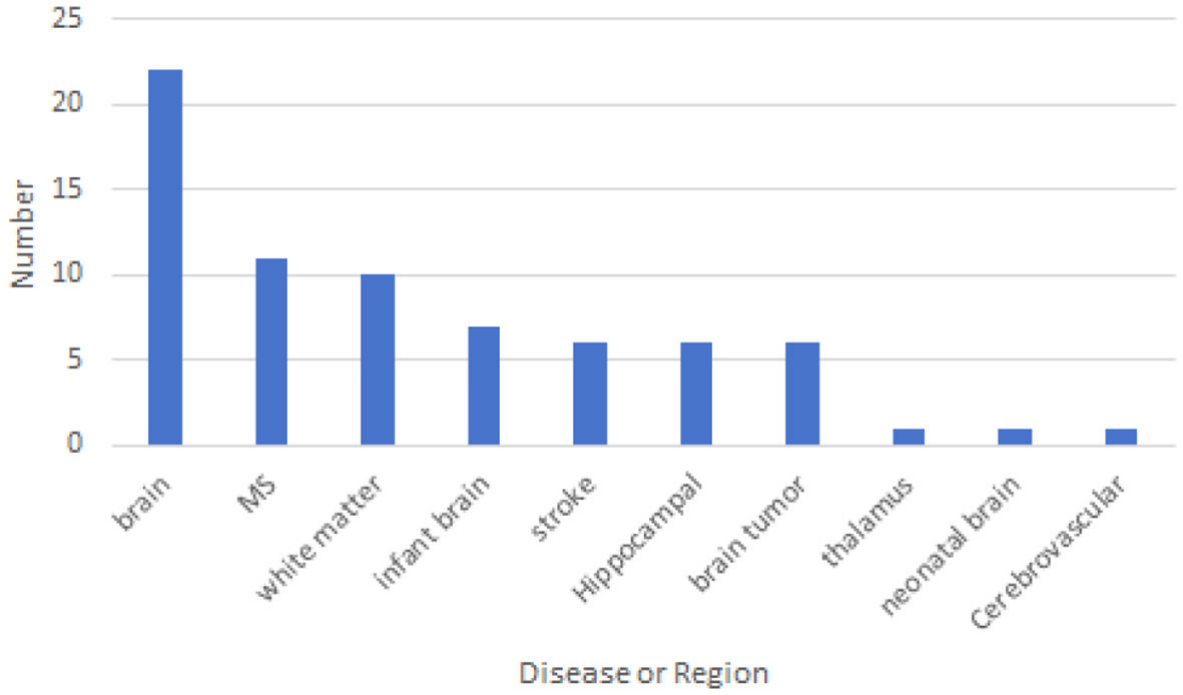
The disease type or brain region that is segmented.

For a more specific analysis, we have included the disease type or brain region that is segmented' in our data extraction. This addition will enable us to gain a deeper understanding of which diseases are related to brain image segmentation and which regions require segmentation. This detailed approach will significantly contribute to our comprehensive review of cross-domain segmentation in brain medical imaging. Figure 6 shows the disease categories and regions extracted from the reviewed papers. Among them, whole-brain segmentation accounts for the largest proportion.

### 3.4 Cross-domain type

**Figure 7:**
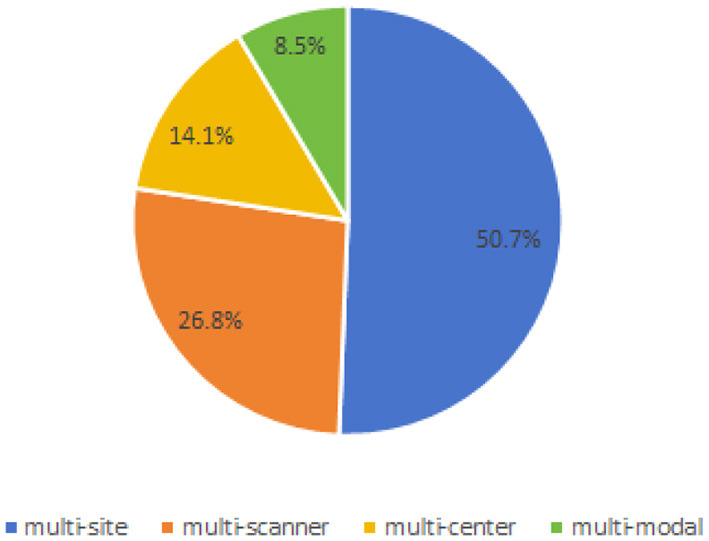
Proportion of cross-domain types.

Based on the data collected, we have identified several types of cross-domain variations present in the field of brain medical image segmentation in Figure 7. The most common type of variation is “multi-site,” with 37 articles addressing this particular challenge. This is followed by “multi-scanner,” which is the focus of 18 articles. Both “multi-center” and “multi-modal” variations were discussed in 10 and six articles each. These findings highlight the diverse range of cross-domain challenges encountered in the segmentation of brain medical images, underscoring the need for further research and method development in this area.

### 3.5 Solution method

**Figure 8:**
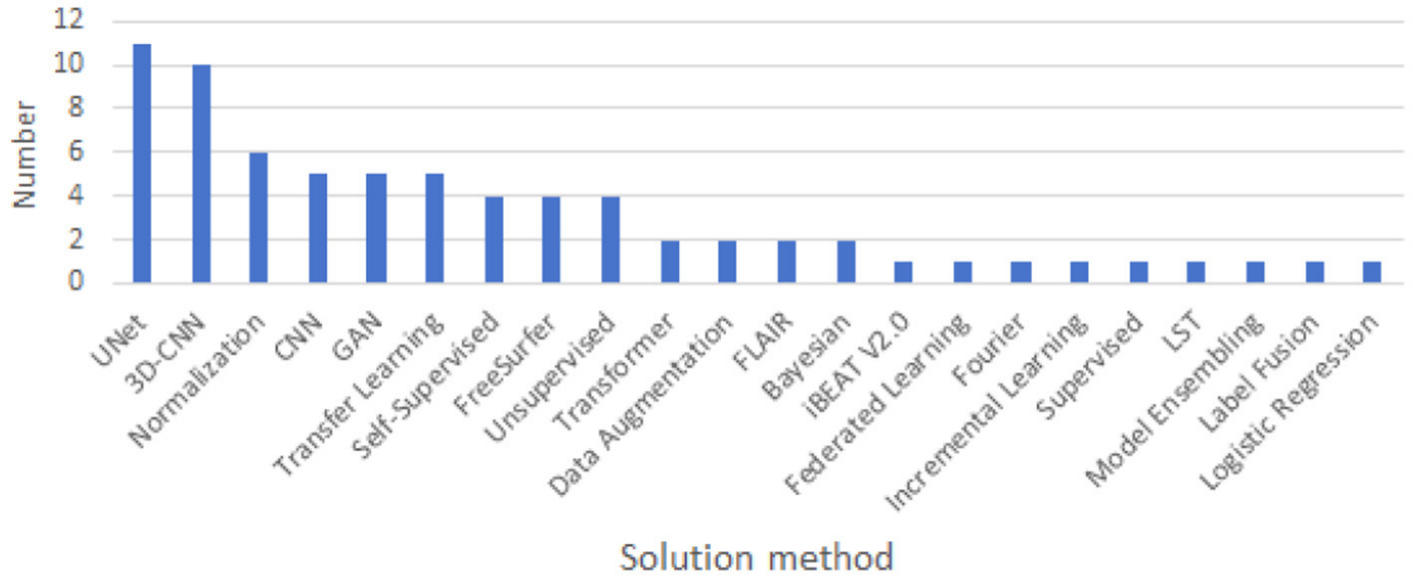
Solution method used for cross-domain.

As show in Figure 8, in the landscape of cross-domain segmentation in brain medical imaging, a diverse range of techniques are employed. The most prevalent methods include UNet, CNN, 3D-CNN, and Transfer Learning, indicating a strong reliance on convolutional architectures and leveraging pre-existing models. Other techniques such as Normalization, Self-Supervised learning, and GANs are also being utilized, albeit less frequently. A handful of studies explore alternative approaches including Unsupervised learning, Data Augmentation, and Transformer-based methods. This diversity of methodologies underscores the complexity of the challenge and the ongoing innovation in the field.

Due to the diversity in datasets and experimental methods, it is not feasible to compare the performance of all algorithms. However, it is possible to compare the algorithms that have utilized the ATLAS, MICCAI 2017 and BraTS datasets.

### 3.6 Stroke lesion segmentation

#### 3.6.1 Dataset

To begin with, we introduce the dataset used, ATLAS. The MR modality of the Anatomical Tracings of Lesions After Stroke (ATLAS) dataset is T1. It has two versions: ATLAS v1.2 (Liew et al., 2017), released in 2018, includes 304 cases from 11 research centers worldwide; and ATLAS v2.0 (Liew et al., 2022), released in 2022, includes 12,71 cases. Although v2.0 contains more data, its relatively recent release means that fewer articles have used it for cross-domain image segmentation to date. Therefore, we have chosen ATLAS v1.2 as our comparison dataset. As shown in Table 5, ATLAS v1.2 includes nine sites.

**Table 5:** The nine source sites of the T1-weighted MR images in experiment.

**Site**	**Location**	**Scanner**	**# Patients**
1	Medical University General Hospital Tianjin, China	GE 750 Discovery	55
2	University of Tübingen Tübingen, Germany	GE Signa Excite	34
3	Sunnaas Rehabilitation Hospital Nesodden, Norway	Siemens Trio	27
4	NORMENT and KG Jebsen Center for Psychosis Research Oslo, Norway	Siemens Trio	12
5	Department of Psychology Oslo, Norway	Phillips Achieva	27
6	Child Mind Institute New York, USA	Siemens Trio	14
7	Nathan S. Kline Institute for Psychiatric Research Orangeburg, USA	Siemens Trio	11
8	University of Texas Medical Branch Galveston, USA	GE 750 Discovery	35
9	University of Michigan Ann Arbor, USA	Siemens Trio	14

#### 3.6.2 Algorithms

Cross-domain algorithms, as the name suggests, are designed to generalize and perform well across multiple, diverse datasets. A notable example from 2023, the Fan-Net (Yu et al., 2023b), utilizes Fourier-based adaptive normalization for stroke lesion segmentation. In 2021, the Unlearning algorithm (Dinsdale et al., 2020) was proposed to unlearn dataset biases for MRI harmonization and confound removal. Similarly, SAN-Net (Yu et al., 2023a) in 2023 and RAM-DSIR (Zhou et al., 2022) in 2022 showcased learning generalization to unseen sites and generalizable medical image segmentation via random amplitude mixup, respectively.

On the other hand, for performance comparison, we have also selected some non-cross-domain algorithms that are optimized for specific tasks or datasets. For instance, U-Net (Ronneberger et al., 2015), proposed in 2015, is an early example of convolutional networks for biomedical image segmentation. In 2018, DeepLab v3+ (Chen et al., 2018) introduced atrous separable convolution for semantic image segmentation. More recently, in 2020, nnU-Net (Isensee et al., 2021) presented a self-configuring method for deep learning-based biomedical image segmentation.

#### 3.6.3 Evaluation result

In the realm of cross-domain segmentation in brain medical imaging, specifically for stroke lesion segmentation, the performance of various methods demonstrates a compelling trend toward the adoption of cross-domain algorithms.

As can be seen from Table 6, Among the non-cross-domain algorithms, CLCI-Net exhibits the highest Dice and F1-score, demonstrating superior performance in segmentation accuracy. However, nnU-Net, despite having a slightly lower Dice score, presents the least Floating Point Operations Per Second (FLOPs), indicating a more efficient use of computational resources.

**Table 6:** Comparison of stroke lesion segmentation method.

**Method type**	**Method**	**DSC**	**Recall**	**F1**	**#Par**	**Mem**	**FLOPs**
	U-net (Ronneberger et al., 2015)	0.471 ± 0.195	0.431 ± 0.193	0.486 ± 0.216	28.94	260.20	31.63
Non-	ResUNet (Zhang et al., 2018)	0.478 ± 0.195	0.469 ± 0.193	0.532 ± 0.184	28.94	260.20	31.63
cross-	Deeplabv3+ (Chen et al., 2018)	0.463 ± 0.207	0.459 ± 0.218	0.471 ± 0.184	59.33	171.63	14.50
domain	nnU-Net (Isensee et al., 2021)	0.504 ± 0.200	0.491 ± 0.199	0.526 ± 0.202	18.67	155.01	10.18
	X-Net (Qi et al., 2019a)	0.508 ± 0.192	0.495 ± 0.184	0.517 ± 0.189	15.05	915.67	20.33
	CLCI-Net (Yang et al., 2019)	0.517 ± 0.192	0.513 ± 0.197	0.512 ± 0.183	36.81	1,235.35	**8.0**
	U-Net3+ (Huang et al., 2020)	0.521 ± 0.207	0.485 ± 0.184	0.497 ± 0.193	26.97	961.57	129.87
	Unlearning (Dinsdale et al., 2020)	0.541 ± 0.188	0.563 ± 0.172	0.536 ± 0.188	27.90	205.73	23.86
Cross-	FAN-Net (Yu et al., 2023b)	0.559 ± 0.180	0.576 ± 0.162	0.545 ± 0.162	28.94	261.59	33.09
domain	DFENet (Basak et al., 2021)	0.530 ± 0.202	0.545 ± 0.187	0.526 ± 0.194	16.72	1,083.52	27.49
	RAM-DSIR (Zhou et al., 2022)	0.556 ± 0.190	0.567 ± 0.183	0.548 ± 0.196	**10.59**	273.24	10.65
	SAN-Net (Yu et al., 2023a)	**0.571** ± 0.195	**0.597** ± 0.158	**0.562** ± 0.192	29.64	**130.79**	33.63

Bold font represents the maximum value.

Shifting focus to cross-domain algorithms, SAN-Net outperforms the rest in all three performance metrics—Dice, Recall, and F1-score, highlighting its robustness in handling cross-domain segmentation tasks. Notably, RAM-DSIR, despite having the least number of parameters, delivers competitive results, suggesting an efficient model with less complexity.

In conclusion, while non-cross-domain algorithms such as CLCI-Net and nnU-Net exhibit commendable performance, cross-domain algorithms, particularly SAN-Net and RAM-DSIR, demonstrate superior performance and efficiency in stroke lesion segmentation. This underscores the potential and advantages of cross-domain approaches in this field, prompting further exploration and development in this direction.

In order to benchmark stroke lesion segmentation algorithms under non-domain adaptation scenarios, we refer to the dataset collated in this study (Malik et al., 2024). As shown in Table 7, eight stroke lesion segmentation algorithms from the ATLAS project were employed. Many of these algorithms achieved a Dice Similarity Coefficient (DSC) of up to 0.7, with the highest-performing algorithm, the seventh one, reaching 0.844. This significantly surpasses the maximum DSC of 0.597 achieved when conducting domain adaptation testing. Therefore, it is currently challenging for domain adaptation algorithms to achieve performance levels comparable to those of algorithms tested without domain adaptation, due to the necessity of conducting domain adaptation testing.

**Table 7:** Stroke lesion segmentation algorithms that do not use cross-domain testing.

**References**	**DSC**	**Pr**	**Re**
Zhang et al. (2021)	0.662	0.694	0.664
Zhou et al. (2019)	0.723	0.633	0.524
Qi et al. (2019b)	0.486	0.6	0.475
Wu et al. (2022)	0.611	0.633	0.676
Hui et al. (2021)	0.592	0.656	0.599
Sheng et al. (2022)	0.556	0.636	0.581
Li (2021)	**0.844**	0.534	–
Wang S. et al. (2022)	0.617	0.63	–

Bold font represents the maximum value.

### 3.7 White matter segmentation

#### 3.7.1 Dataset

As shown in Table 8, the dataset MICCAI 2017 is derived from the WMH MICCAI 2017 challenge (Kuijf et al., 2019). This dataset encompasses MRI scans from multiple sites, including the University Medical Center Utrecht (UMC Utrecht), the National University Health System Singapore (NUHS Singapore), the VU University Medical Center Amsterdam (VU Amsterdam), and two undisclosed locations.

**Table 8:** WMH segmentation MICCAI 2017 challenge dataset.

**Site**	**Location**	**Scanner**	**T1 voxel size (*mm*^3^)**	**FLAIR scans size (*mm*^3^)**	**Train**	**Test**
1	UMC Utrecht	3T Philips Achieva	1.00*1.00*1.00	0.96*0.95*3.00	20	30
2	NUHS Singapore	3T Siemens TrioTim	1.00*1.00*1.00	1.00*1.00*3.00	20	30
3	VU Amsterdam	3T GE Signa HDxt	0.94*0.94*1.00	0.98*0.98*1.20	20	30
4	Unknown	1.5T GE Signa HDxt	0.98*0.98*1.50	1.21*1.21*1.30	0	10
5	Unknown	3T Philips Ingenuity	0.87*0.87*1.00	1.04*1.04*0.56	0	10

The MRI scans in the dataset are obtained from a variety of scanners, including 3T Philips Achieva, 3T Siemens TrioTim, 3T GE Signa HDxt, 1.5T GE Signa HDxt, and 3T Philips Ingenuity. The T1 voxel sizes and FLAIR scan sizes captured by these scanners vary, ranging from 0.87*0.87*1.00 *mm*^3^ to 1.21*1.21*1.30 *mm*^3^.

In total, 60 samples are utilized for training, while the testing set comprises 110 samples. The diversity and scale of this dataset allow us to evaluate the performance of our methods in a comprehensive and accurate manner. The training data can be downloaded at https://wmh.isi.uu.nl.

#### 3.7.2 Algorithms

In the context of white matter medical imaging, several notable papers stand out. The Voxel-Wise Logistic Regression (VLR) (Knight et al., 2018) algorithm, introduced in 2018, leveraged voxel-wise logistic regression for FLAIR-based white matter hyperintensity segmentation. An innovative approach was presented in 2019 with the Skip Connection U-net (SC U-net) (Zhang et al., 2019), which added skip connections to the classic U-net architecture. In 2021, the MixDANN (Kushibar et al., 2021) algorithm tackled the challenging scenario of domain generalization (DG), i.e., training a model without any knowledge about the test distribution. The same year, an Ensemble U-net (Park et al., 2021) with multi-scale highlighted foreground (HF) was introduced for white matter hyperintensity segmentation, demonstrating its effectiveness in cross-domain segmentation in the 2017 MICCAI white matter hyperintensity segmentation challenge. A Transductive Transfer Learning Approach (TDA) (Kruger et al., 2021) was proposed in 2021 for domain adaptation, aiming to reduce the domain shift effect in brain MRI segmentation.

#### 3.7.3 Evaluation result

Table 9 presents the results of five different methods, all of which focus on the cross-domain segmentation problem in white matter imaging. In the table, – means there is no valid data. However, it is important to note that, with the exception of the second and third methods, the experimental datasets and experimental procedures used in each method are distinct from each other.

**Table 9:** Comparison of white matter segmentation method.

**Method**	**Dataset name**	**Site number**	**Data number**	**DSC**	**Recall**	**F1**
VLR (Knight et al., 2018)	MICCAI 2017, MICCAI 2016, ISBI MS 2015	7 = 3 + 3 + 1	96 = 3*20 + 3*5 + 21	0.70	0.78	–
SC UNet (Zhang et al., 2019)	MICCAI 2017	3	60 = 3*20	0.78	–	–
MixDANN (Kushibar et al., 2021)	MICCAI 2017	3	60 = 3*20	0.74	0.69	0.66
ensemble UNet (Park et al., 2021)	MICCAI 2017	5	170 = 3*50 + 2*10	0.81	0.82	0.79
TDA (Kruger et al., 2021)	MICCAI 2017, VH	4 = 3 + 1	88 = 3*30 + 28	0.59	0.51	–

For instance, the VLR method employed three datasets, which included seven sites, and performed a leave-one-out cross-validation with respect to these sites. The SC U-net and MixDANN methods, on the other hand, only employed three sites from the MICCAI 2017 training data for cross-validation. The Ensemble U-net method used all of the training data from MICCAI 2017 for training and the test data for testing. Lastly, the TDA method utilized both the MICCAI 2017 and VH datasets, performing cross-validation between these datasets. In addition, VH is a private dataset.

Therefore, while there are numerous studies addressing the cross-domain problem in the field of white matter segmentation, direct comparisons between them are challenging. This is due to the variations in the experimental data and procedures used, even when the same dataset is utilized in different studies. The differences in experimental procedures are manifested in whether cross-validation is performed between sites or between datasets.

Although it is challenging to make a direct comparison between each algorithm, an overall observation can be made in the field of white matter segmentation. Specifically, the Dice Similarity Coefficient (DSC) is above 0.7 when cross-validation is conducted between sites, while the DSC is only around 0.5 when cross-validation is carried out between datasets. This observation suggests that cross-validation between datasets is more challenging, yet it is also closer to real-world scenarios.

### 3.8 Brain tumor segmentation

#### 3.8.1 Dataset

In Table 10, the BraTS datasets comprises three dataset: BraTS 2015, BraTS 2018, and BraTS 2019, each with varying numbers of cases. The datasets are categorized into two major classes: High-Grade Gliomas (HGG) and Low-Grade Gliomas (LGG). Each case consists of four modalities (T1, T2, FLAIR, T1ce) and requires segmentation into three parts: Whole Tumor (WT), Enhancing Tumor (ET), and Tumor Core (TC). The BraTS 2019 can be downloaded at https://www.med.upenn.edu/cbica/brats-2019/.

**Table 10:** BraTS dataset.

**Dataset name**	**Site number**	**HGG number**	**LGG number**
BraTS 2015 (Menze et al., 2015)	–	220	54
BraTS 2018 (Bakas et al., 2018)	19	210	75
BraTS 2019	19	259	76

#### 3.8.2 Algorithms

In 2021, a learnable Self-Attentive Spatial Adaptive Normalization (SASAN) (Tomar et al., 2021) method was introduced, utilizing adversarial training to address the domain gap in radiological images. In 2022, two algorithms were presented. One algorithm is grounded in a knowledge distillation scheme incorporating exponential mixup decay (EMD) (Liu et al., 2022b) to progressively acquire target-specific representations, while the other algorithm is the Unsupervised Domain Adaptation (UDA) method based on Self-Semantic Contour Adaptation (SSCA) (Liu et al., 2022a). In 2023, another UDA (Qin et al., 2023) method, based on semi-supervised learning, was proposed. Additionally, in the same year, the Multimodal Contrastive Domain Sharing (Multi-ConDoS) (Zhang et al., 2023) generative adversarial networks were introduced.

#### 3.8.3 Evaluation result

As shown in Table 11, Whole, Core, and Enh represent the Dice Similarity Coefficient (DSC) for whole tumor, core tumor, and enhanced tumor, respectively. While all five articles conducted cross-domain studies on brain tumor segmentation using the BraTS datasets, each article employed different source and target domains. As a result, direct comparisons of algorithm performance across the experimental results are challenging.

**Table 11:** Comparison of brain tumor segmentation method.

**Method**	**Dataset name**	**Source domain**	**Target domain**	**Source to target**	**Whole**	**CoreT**	**EnhT**
SSCA (Liu et al., 2022a)	BraTS 2018	285	285	T2 to T1, T1ce, FLAIR	0.68	0.58	0.45
MultiConDoS (Zhang et al., 2023)	Hecktor, BraTS 2018	201	210	CT to MRI	0.58	–	–
UDA (Qin et al., 2023)	BraTS 2019	335*2	335*2	T1 + T1ce to T2 + FLAIR	0.49	0.31	0.22
SASAN (Tomar et al., 2021)	WHSD, BraTS 2015	20	65	T2 to T1	0.61	0.18	0.46
EMD (Liu et al., 2022b)	BraTS 2018	210	75	HGG to LGG	0.83	0.46	0.32

## 4 Discussion

The field of brain medical image segmentation has seen significant advancements with the widespread application of deep learning technologies. However, the challenge of domain adaptation continues to be a crucial issue. In our review, we have identified a variety of methods proposed to address this issue, including transfer learning, normalization, unsupervised learning, Transformer models, and convolutional neural networks, among others. Each of these methods has its strengths but also comes with certain limitations.

Transfer learning is a common approach to addressing domain adaptation issues, with the main idea being to apply knowledge learned in one domain (source domain) to another domain (target domain). However, the effectiveness of this method is influenced by the distribution difference between the source and target domains. If the distribution difference is too large, the effectiveness of transfer learning may be compromised.

Normalization is another common method for addressing domain adaptation issues, with the main idea being to reduce the differences between different datasets by adjusting the brightness and contrast of images. However, this method may result in the loss of some important image information, thereby affecting the accuracy of segmentation results.

Unsupervised learning and Transformer models have also been used in some studies to address domain adaptation issues. The advantage of unsupervised learning is that it does not require labeled data, but its performance is usually not as good as supervised learning. The advantage of Transformer models is that they can handle long-distance dependencies, but they have a high computational complexity and require a large amount of computational resources.

Furthermore, we have observed that despite the application of various techniques to address domain adaptation issues in brain medical imaging, there currently exists a lack of unified dataset collections and experimental standards.

For instance, as illustrated in Figure 4, 42.3% of the papers only use private data, while 8.5% of the papers use both public and private data. As shown in Figure 7, even when public datasets are used, there is significant diversity amongst them. As indicated in Tables 9, 11, even when a single identical dataset is used, if the experimental data and methods differ, it remains challenging to make comparisons among various algorithms. Moreover, the vast majority of current algorithms are not open-source, making it nearly impossible to reproduce the algorithms in the papers and design similar experiments for comparison.

Consequently, this makes it difficult to compare the performance of different studies and accurately assess the effectiveness of new methods. Therefore, future research needs to further develop more effective domain adaptation methods and establish unified dataset collections and experimental standards.

## 5 Conclusions

In conclusion, domain adaptation in brain medical image segmentation is a challenging research field that necessitates further exploration and development. Although numerous methods have been proposed to tackle this issue, each possesses its own strengths and limitations. Future research needs to delve deeper into novel methods to enhance the performance of domain adaptation in brain medical image segmentation.

Moreover, it is imperative to establish unified dataset collections and experimental standards for a more accurate evaluation of the performance of different methods. Only through this approach can we gain a better understanding of the strengths and weaknesses of various methods and develop more effective solutions.

Finally, we anticipate further advancements in deep learning technologies to address the domain adaptation problem in brain medical image segmentation. This progress will improve the accuracy of medical image analysis and, ultimately, enhance patient diagnosis and treatment.

## Data availability statement

The original contributions presented in the study are included in the article/supplementary material, further inquiries can be directed to the corresponding author.

## Author contributions

MY: Writing – original draft, Writing – review & editing, Investigation, Methodology, Validation. CS: Data curation, Investigation, Methodology, Software, Writing – review & editing. LW: Data curation, Investigation, Validation, Visualization, Writing – review & editing. YZ: Formal analysis, Investigation, Validation, Writing – review & editing. AW: Funding acquisition, Project administration, Resources, Supervision, Writing – review & editing.
